# Supplementary material for: Vitamin A Promotes the Fusion of Autophagolysosomes and Prevents Excessive Inflammasome Activation in Dextran Sulfate Sodium-Induced Colitis
Source: Int J Mol Sci. 2023 May 12;24(10):8684. doi: 10.3390/ijms24108684 (PMC10218524; doi:10.3390/ijms24108684)
Supplement: Supplementary file 1 [file ijms-24-08684-s001.zip › ijms-2342145-supplementary.pdf]

SUPPLEMENTAL INFORMATION

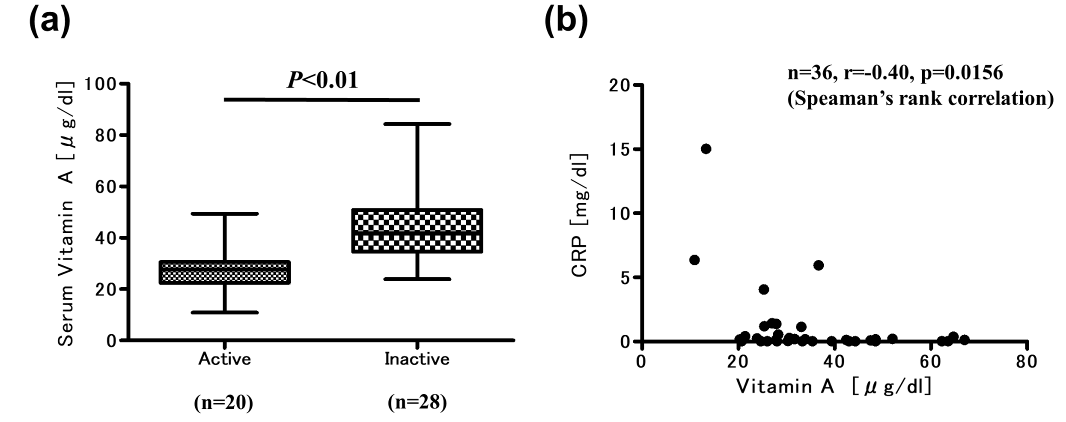

Figure S1. Serum vitamin A levels of patients with CD are related to disease activity and CRP.

(A) Serum vitamin A levels of active CD (Crohn's disease activity index (CDAI) <150, n=20) and inactive CD (CDAI ≥150, n=28) patients, (B) correlation between serum vitamin A levels and C-reactive protein (CRP) of CD patients (n=36). In (A), data are presented as mean ± SEM (n>2 independent experiments). Unpaired-*t* test was used for statistical analysis (**P*<0.05, ***P*<0.01). In (B), data was analyzed with Speaman's rank correlation.

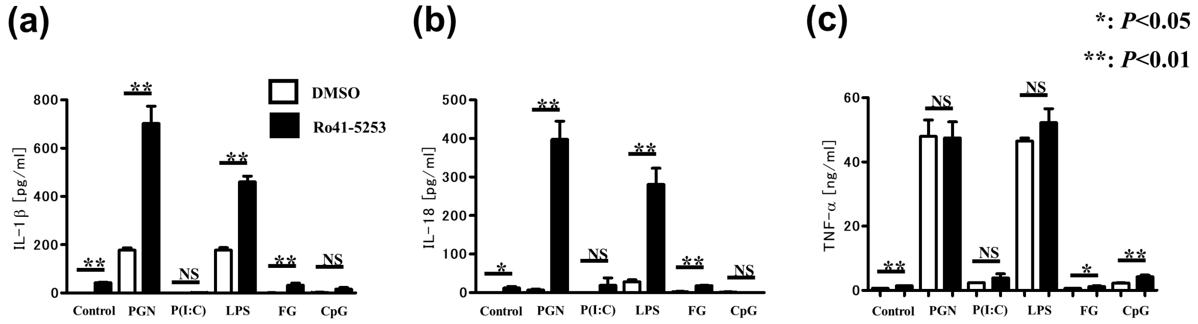

Figure S2. RAW 264.7 cells pretreated with RAR antagonist produce increased levels of IL-1β and IL-18. (A, B, C) Effect of a RAR antagonist (Ro41-5253) on inflammatory cytokine production of murine macrophage (RAW264.7) cells treated with TLR ligands. RAW264.7 cells were cultured in the presence of TLR ligands (PGN, Poly(I:C), LPS, Flagellin, CpG) for 24 h, pre-treated with or without a RAR antagonist (Ro41-5253) for 1 h. Inflammatory cytokines (IL-1β, IL-18, TNF-α) were measured in culture supernatants by ELISA. (A) IL-1β production (n=5), (B) IL-18 production (n=5), (C) TNF-α production (n=5). In (A, B, C), data are presented as mean ± SEM (n>2 independent experiments). Unpaired-*t* test was used for statistical analysis (*P<0.05, **P<0.01).

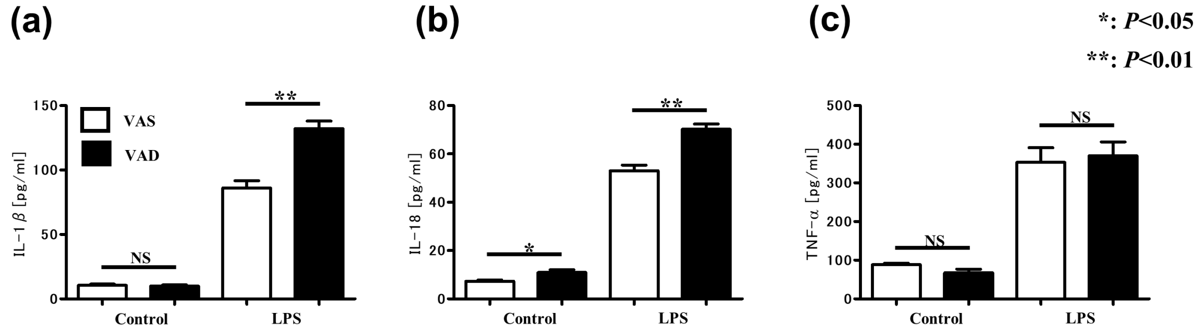

Figure S3. LPS-treated VAD CD11b+ splenic cells exhibit markedly increased levels of IL-1β and IL-18. (A, B, C) Inflammatory cytokine production of $CD11b^{+}$ splenic cells from VAS and VAD B6 mice treated with LPS. Inflammatory cytokines (IL-1β, IL-18, TNF-α) were measured in culture supernatants by ELISA. (A) IL-1β production (n=5), (B) IL-18 production (n=5), (C) TNF-α production (n=5), In (A, B, C), data are presented as mean ± SEM (n>2 independent experiments). Unpaired-*t* test was used for statistical analysis (*P<0.05, **P<0.01).

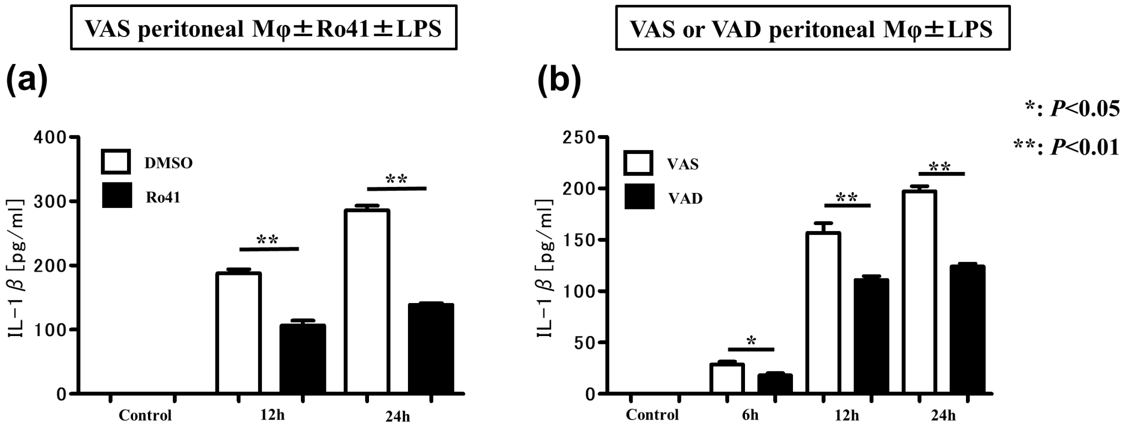

Figure S4. LPS-treated VAD B6 peritoneal macrophages produce markedly reduced levels of IL-1β and IL-18. (A, B) IL-1β production of PECs, (A) IL-1β production of PECs collected from VAS and VAD mice after LPS treatment (n=5), (B) IL-1β production of PECs collected from VAS mice pre-treated with or without a RAR antagonist (Ro41-5253) after LPS treatment (n=5). IL-1β were measured in culture supernatants by ELISA. In (A, B), data are presented as mean ± SEM (n>2 independent experiments). Unpaired-*t* test was used for statistical analysis (**P*<0.05, ***P*<0.01).

SUPPLEMENTAL EXPERIMENTAL PROCEDURES

Preparation of $CD11b^+$ splenic cells

Single-cell suspensions from spleens of mice were prepared as previously described [89]. Cells were purified by AutoMACS with CD11b microbeads (Miltenyi Biotec Inc., Bergish Gladbach, Germany). More than 95% of the recovered cells were $CD11b^+$. Splenic $CD11b^+$ cells were resuspended in RPMI 1640 medium supplemented with 10% FBS and seeded on 24-well culture plates at 1×10^6 cells/well. Cells were treated with 10 μg/ml LPS.

Preparation of peritoneal exudate cells (PECs)

Mice were injected with 3 ml of 4% thioglycolate (Eiken Chemical Co., Ltd., Tokyo, Japan) intraperitoneally. After 4 days, mice were injected intraperitoneally with 6 ml cold PBS and peritoneal exudate cells (PECs) were harvested. PECs were seeded on 24-well culture plate at 1×10^6 cells/well in RPMI 1640 medium (Thermo Fisher Scientific) supplemented with 0.1 mg/ml recombinant macrophage colony-stimulating factor (PeproTech, Rochy Hill, USA) and 10% FBS and incubated overnight [90]. Cells were then treated with 10 mM Ro41-5253 or DMSO as control group for 1 h and treated simultaneously with 10 μg/ml LPS.
